# Recent Advances in Nanomaterials for Modulation of Stem Cell Differentiation and Its Therapeutic Applications

**DOI:** 10.3390/bios14080407

**Published:** 2024-08-22

**Authors:** Chang-Dae Kim, Kyeong-Mo Koo, Hyung-Joo Kim, Tae-Hyung Kim

**Affiliations:** School of Integrative Engineering, Chung-Ang University, 84 Heukseuk-ro, Dongjak-gu, Seoul 06974, Republic of Korea; rlackdeo4@cau.ac.kr (C.-D.K.); sse0913@cau.ac.kr (K.-M.K.); zoorry@cau.ac.kr (H.-J.K.)

**Keywords:** nanomaterials, intracellular regulation, cellular adhesion, external stimulation, stem cell differentiation

## Abstract

Challenges in directed differentiation and survival limit the clinical use of stem cells despite their promising therapeutic potential in regenerative medicine. Nanotechnology has emerged as a powerful tool to address these challenges and enable precise control over stem cell fate. In particular, nanomaterials can mimic an extracellular matrix and provide specific cues to guide stem cell differentiation and proliferation in the field of nanotechnology. For instance, recent studies have demonstrated that nanostructured surfaces and scaffolds can enhance stem cell lineage commitment modulated by intracellular regulation and external stimulation, such as reactive oxygen species (ROS) scavenging, autophagy, or electrical stimulation. Furthermore, nanoframework-based and upconversion nanoparticles can be used to deliver bioactive molecules, growth factors, and genetic materials to facilitate stem cell differentiation and tissue regeneration. The increasing use of nanostructures in stem cell research has led to the development of new therapeutic approaches. Therefore, this review provides an overview of recent advances in nanomaterials for modulating stem cell differentiation, including metal-, carbon-, and peptide-based strategies. In addition, we highlight the potential of these nano-enabled technologies for clinical applications of stem cell therapy by focusing on improving the differentiation efficiency and therapeutics. We believe that this review will inspire researchers to intensify their efforts and deepen their understanding, thereby accelerating the development of stem cell differentiation modulation, therapeutic applications in the pharmaceutical industry, and stem cell therapeutics.

## 1. Introduction

Stem cells are a versatile and promising class of cells that can self-renew and differentiate into various specialized cell types, which makes them a valuable tool for regenerative medicine and tissue engineering [1,2,3,4,5,6]. For example, stem cell therapy offers a new paradigm for individuals with untreatable conditions, shifting the focus of treatment from solely managing the disease to modulating immunopharmacological intervention and regeneration [7,8,9,10,11]. In recent years, research on stem cells has produced increasing evidence suggesting that stem cell transplantation is a highly effective approach for treating neurological disorders, bone injuries, and various diseases [12,13,14,15,16,17,18]. However, stem cell transplantation for clinical use has limited effectiveness in producing mature specialized cells to replace damaged cells [19,20,21]. In contrast, ex vivo differentiation of stem cells is known to have low efficiency and poor survival when transplanted into the body [22,23]. Moreover, the ability to differentiate stem cells into specific cell types of interest (e.g., bones, cartilage, and muscles) in a highly selective and efficient manner remains a significant challenge [24,25,26,27,28]. To fully realize the therapeutic potential of stem cells in the field of regenerative medicine, precise control of the fate of stem cells should be addressed [29,30,31,32,33,34,35,36,37]. The surrounding matrix can significantly influence the development and specialization of stem cells. Moreover, altering factors such as the size, hydrophilicity, roughness, and organization of the cell attachment surface can directly affect the cellular activity.

The field of nanotechnology has made significant advancements in influencing the stem cell behavior through the application of various types of nanomaterials, including metal- and carbon-based ones and nanoframeworks [38,39,40,41,42,43,44,45]. Metallic nanomaterials, such as gold nanoparticles (AuNPs), silver nanoparticles (AgNPs), and other metal-based nanoparticles, have recently gained significant attention owing to their wide range of applications, including reactive oxygen species (ROS) scavenging, autophagy, and thermoplasmonic regulation [46,47,48,49,50,51,52]. Carbon-based nanomaterials encompass fullerenes, carbon nanotubes (CNTs), graphene and its derivatives, graphene oxide (GO), nanodiamonds (NDs), and carbon-based quantum dots (CQDs) [53,54,55,56,57,58]. These materials have attracted significant interest because of their distinct structural dimensions and remarkable properties in biomedical fields, including cancer therapy and wearable device (reviewed elsewhere [59,60]). Finally, we focused the practicality of different nanomaterials in regulating biomolecule delivery and facilitating stem cell specialization, focusing on metal–organic frameworks (MOFs), zeolite imidazolate frameworks (ZIFs), and upconversion nanoparticles (UCNPs) in stem cell therapies [61,62]. In particular, nanomaterials with biodegradable and biocompatible properties can be engineered to mimic the natural extracellular matrix and provide specific chemical and physical cues to guide stem cell differentiation and proliferation [63,64].

Recent studies have investigated whether nanostructured surfaces and scaffolds can enhance the proliferation, migration, and differentiation of stem cells into specific lineages, such as osteogenic, adipogenic, or neurogenic differentiation [65,66,67]. For instance, the surface topography, stiffness, and chemical composition of nanomaterials have been shown to significantly impact stem cell differentiation [68]. Moreover, nanomaterials can serve as delivery vehicles (e.g., metal–organic framework; self-assembled, peptide-based nanodrugs) for various bioactive molecules, growth factors, and genetic materials, which further enhances stem cell differentiation and tissue regeneration [69,70,71]. This allows for the precise control and guidance of stem cell differentiation, which is crucial for the development of stem-cell-based therapies. The increased use of nanostructures in stem cell research has led to the development of several new technologies, highlighting a substantial demand for innovative therapeutic approaches.

**Figure 1:**
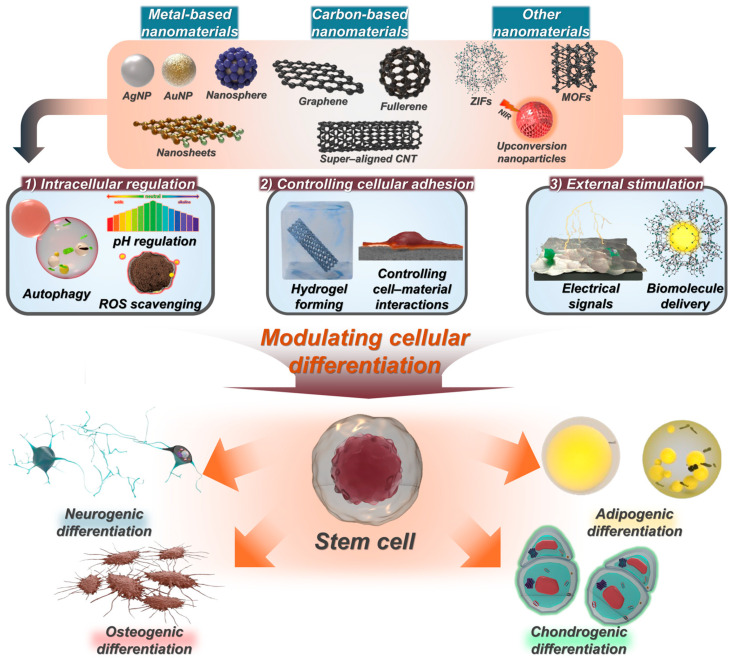
Schematic illustrations of various nanomaterials to modulate stem cell functions and mechanisms.

This review provides an overview of recent advances in nanomaterials across metal-, carbon-, and peptide-based approaches, focusing on their applications in enhancing stem cell differentiation and therapeutic strategies (Figure 1). It also focuses on strategies that commonly integrate nanostructures to enhance differentiation and healing efficiency, along with descriptions of common nanomaterial fabrication approaches used in stem cell research. Finally, we conclude with a future perspective highlighting clinical applications of stem cell therapy and advancement in point-of-care treatments.

## 2. Metal-Based Stem Cell Differentiation Approaches and Therapeutics

Metallic nanoparticles can guide stem cell fate and influence their proliferation, migration, and differentiation. Nanomaterials such as gold nanoparticles (AuNPs), silver nanoparticles (AgNPs), and other metal-based nanoparticles have recently attracted considerable attention for potent and broad applications such as medical carriers for in regenerative medicine. These nanoparticles are used to direct stem cell differentiation toward desired lineages, thereby enhancing the therapeutic potential of these cells. The applications of metal-based nanomaterials in stem cell research extend beyond directing differentiation; they also show promise in stem cell tracking and imaging [46,72,73]. Moreover, the integration of metal-based nanomaterials with advanced biomaterials, such as hydrogels and diverse scaffolds, has further expanded the therapeutic potential of these systems [74,75]. Here, we describe the use of various metal-based nanomaterials in controlling stem cell fate and biomedical applications. (Table 1).

### 2.1. Autophagy

Numerous studies have demonstrated that the autophagy process is crucial for preserving cellular homeostasis and enabling differentiation under adverse conditions [85,86,87]. Additionally, impairment of cellular autophagy can lead to metabolic disorders, such as accumulation of damaged proteins and/or organelles and inability to clear protein aggregates, leading to compromised stemness and regenerative capacity of stem cells [88,89]. Recent studies have shown that specific types of nanomaterials can be internalized in cells and can accumulate in cellular compartments, including endosomes, lysosomes, and autophagosomes, which activates autophagy through their biological effects [90]. Accordingly, for therapeutic applications, nanoparticles can be used to target the autophagy–lysosome system for stem cell rejuvenation. AuNPs have emerged as the most preferred type of nanoparticle for use in biological and pharmaceutical applications because of their unique surface plasmon resonance and optical properties, as along with their easily modifiable size, shape, functionalization, biocompatibility, and regenerative ability [91,92,93].

**Figure 2:**
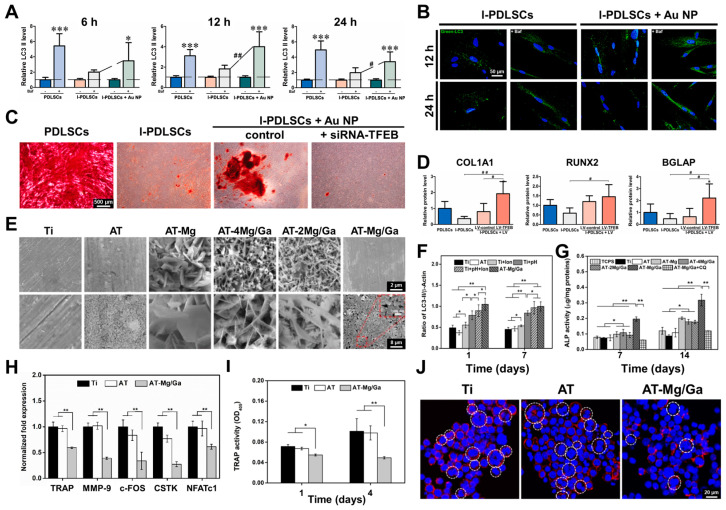
(**A**) AuNP treatment enhanced autophagic activity in inflammatory-conditioned periodontal ligament stem cells (I-PDLSCs) during the early phase of osteogenic differentiation represented by upregulated levels of LC3 II in the AuNP-treated I-PDLSCs and control PDLSCs. (**B**) Confocal images of accumulated FITC-LC3 puncta per cell in the AuNP-incubated I-PDLSCs and I-PDLSCs. (**C**) Knockdown of TFEB abrogated the AuNP-mediated rescue of the osteogenic potential of I-PDLSC. (**D**) Osteogenic protein expression in the I-PDLSCs represented by the decrease in RUNX2 expression. (**E**) Topographic images of different Ti substrates using SEM. (**F**) Quantitative analysis of the LC3 expression in MSCs treated with various samples in normal DMEM media at 1 and 7 days. (**G**) Related qualitative ALP activity on various sample surfaces at 7 and 14 days. (**H**) Relative mRNA expression of osteoclastogenesis-related genes in the RAW264.7 cells grown on different substrates. (**I**) quantitative TRAP activities after incubation for 1 and 4 days. (**J**) Confocal images of multinucleated cells on different substrates after culturing for 4 days. The asterisks and number signs indicate *p*-values *p and ^#^ *p* < 0.05, ** *p* and ^##^ *p* < 0.01, and *** *p* < 0.001. Reprinted with permission from [94]. Copyright 2022, Elsevier; reprinted with permission from [97]. Copyright 2022, Elsevier.

Yin et al. explored the potential of AuNPs to mitigate inflammation-compromised osteogenic differentiation in the periodontal ligament stem cells (PDLSCs) [94]. They evaluated the influence of AuNPs with different particle sizes on the viability and osteogenic differentiation of the PDLSCs and their inflammatory conditions (I-PDLSC). In terms of autophagy, the AuNP treatment did not change the expression of LC3 II, an indicator of the autophagic level, in I-PDLSCs during the early stage of osteogenic differentiation. This was indicated by the lack of significant difference in the LC3 II levels between the AuNPs and I-PDLSCs group. Moreover, the AuNP treatment did not change the expression of LC3 II in the I-PDLSCs during the early stage of osteogenic induction, as indicated by the lack of a significant difference in the LC3 II levels between the AuNPs and I-PDLSCs group. In contrast, the AuNP treatment increased the autophagic flux in the I-PDLSCs, as indicated by the significantly increased accumulation of LC3 II observed in the AuNP-treated I-PDLSCs compared to the I-PDLSCs group at 12 or 24 h during the early stage of osteogenic differentiation. Furthermore, quantification of cellular autophagosomes revealed an elevated proportion of RFP⁺-GFP⁺-LC3 puncta in the AuNP-treated I-PDLSCs following treatment with Bafilomycin A1 (Baf), an autophagy inhibitor, treated at 12 and 24 h during osteogenic differentiation. This is consistent with the enhanced autophagic flux observed in these cells (Figure 2A). However, the Baf treatment only enhanced the proportion of autophagosomes (RFP⁺-GFP⁺-LC3 puncta) in the I-PDLSCs during osteogenic differentiation. Moreover, the AuNP incubation increased the number of accumulated FITC-labeled LC3 puncta in the I-PDLSCs compared to that in the control I-PDLSCs group (Figure 2B). The activation of transcription factor EB (TFEB), a master regulator of the autophagy–lysosome system, and the expression of autophagy- or lysosome-related genes in I-PDLSCs were also examined after the AuNP treatment. The nuclear localization of the TFEB was enhanced in both the I-PDLSCs and AuNP-treated I-PDLSCs after osteogenic induction. However, the AuNP-treated I-PDLSCs exhibited greater nuclear TFEB levels during the osteogenic differentiation than the control I-PDLSCs. Specifically, the knockdown of the TFEB in the AuNP-treated I-PDLSCs inhibited the AuNP-induced restoration of mineralized nodule formation (Figure 2C) and osteogenesis-related protein expression (Figure 2D).

In organ transplantation, often the final therapeutic option in severe diseases, survival is often limited by immunogenic rejection and/or bacterial infection [95]. Utilizing gallium (Ga) coatings on biomedical devices can leverage their antibacterial properties, affecting various bacteria [96]. Chen et al. developed a series of Mg-Ga-layered double hydroxide (LDH) nanosheets on alkaline-treated titanium surfaces, which are composed of positively charged brucite-like layers [97]. They fabricated Mg/Ga LDH sheets on the surface of alkali-heat-treated titanium (AT) implants, which were subsequently calcinated to convert them into Mg/Ga-layered double-oxide nanosheets with enhanced alkalinity and stability. Their aim was to develop bone repair biomaterials and investigate the relationship between autophagy and the pH of the local microenvironment. Therefore, enhancing the alkalinity of implant biomaterial surfaces may prove to be an effective strategy to enhance autophagy and favor osteogenesis under osteoporotic conditions.

Through biological investigations, researchers discovered that the coating could promote autophagy by increasing the alkalinity of the surrounding environment, thereby facilitating the osteogenic differentiation of mesenchymal stem cells (MSCs) and inhibiting the bone resorption activity of osteoclasts. Scanning electron microscopy (SEM) analysis revealed the surface topographies of the samples (Figure 2E); the pure titanium and AT substrates exhibited relatively smooth surface morphologies. The Mg^2+^ and Ga^3+^ ions were incorporated into Mg-exchanged substrates through a high-pressure hydrothermal process and subsequent calcination. The resulting surface topography of the coatings displayed sheet-like structures, with the sizes of these sheets progressively decreasing from the AT-Mg to AT-Mg/Ga samples as the amount of Ga^3+^ increased. Further, to investigate the influence of pH, metal ions, and substrate topography on autophagic activity, an autophagosome formation test was performed on MSC under various conditions. The results (Figure 2F) indicate that the presence of the Mg and Ga metal ions and the topography of AT-Mg/Ga had no significant effect on the level of autophagy. However, the expression level of LC3 II in the pH 8.5 group was higher than in other groups. Interestingly, the addition of an autophagy inhibitor eliminated the differences in ALP activity and mineralization between the AT-Mg/Ga group and the control groups (Figure 2G). Subsequently, osteoclastogenesis-related genes in RAW264.7 cells were analyzed via qRT-PCR under the influence of the receptor activator of the nuclear factor kappa-B ligand (RANKL) and macrophage colony-stimulating factor (m-CSF). As shown in Figure 2H, the AT-Mg/Ga group exhibited significantly lower expression of osteoclastogenesis-related genes compared to the Ti and AT groups. To evaluate osteoclast responses, researchers quantified the tartrate-resistant acid phosphatase (TRAP) activity, a histochemical marker for osteoclasts, in the RAW264.7 cells cultured on three sample groups under the influence of RANKL and m-CSF. After 1 and 4 days of culture, the TRAP activity was significantly lower in the AT-Mg/Ga group than the control groups, particularly at day 4 (Figure 2I). Additionally, while the AT-Mg/Ga sample exhibited only a few multinuclear cells, numerous such cells were readily observed on the Ti and AT groups (Figure 2J). The results indicated that AT-Mg/Ga had significant potential to inhibit the differentiation of the RAW264.7 cells in vitro and suppress osteoclast generation and osteoclastic bone resorption in vivo under osteoporotic conditions. This suggests that AT-Mg/Ga materials could be applied in the development and research of functional orthopedic implants for patients with osteoporosis.

In conclusion, metal nanostructures, particularly AuNPs and GaNPs, have proven to be crucial for preserving the potency and enhancing the differentiation capacity of undifferentiated stem cells. Such metal-based nanomaterials are extensively utilized to facilitate and promote stem cell differentiation across diverse applications.

### 2.2. ROS Scavenger

Stem-cell-based tissue regeneration has emerged as a promising approach for the treatment of severe traumatic injuries and chronic wounds, including cardiac repair, neurological trauma, bone defects, cartilage damage, and diabetic foot complications. However, the low survival and impaired function of implanted stem cells, largely due to excessive ROS in the damaged microenvironment, have significantly limited their therapeutic efficacy [98]. To address this challenge, engineered antioxidant nanomaterials have been explored as potential strategies to enhance the resistance of stem cells to oxidative stress and promote their regenerative capacity. Accordingly, the development of ROS-scavenging nanostructures has emerged as an intriguing approach to protect and regulate stem cells, thereby facilitating tissue regeneration in high-ROS environments [99,100]. For instance, selenium, silver, and aluminum nanoparticles have been utilized to alleviate oxidative stress and enhance the stemness and stem cell differentiation. While recent progress has been made in developing catalytic materials that can scavenge ROS, designing high-performance, broad-spectrum ROS-scavenging materials with rapid enzyme-like catalytic kinetics remains a significant challenge. Therefore, it is crucial to develop suitable strategies to address the imbalanced valence states during catalytic ROS scavenging and achieve reversible catalytic cycles with high reaction activities.

**Figure 3:**
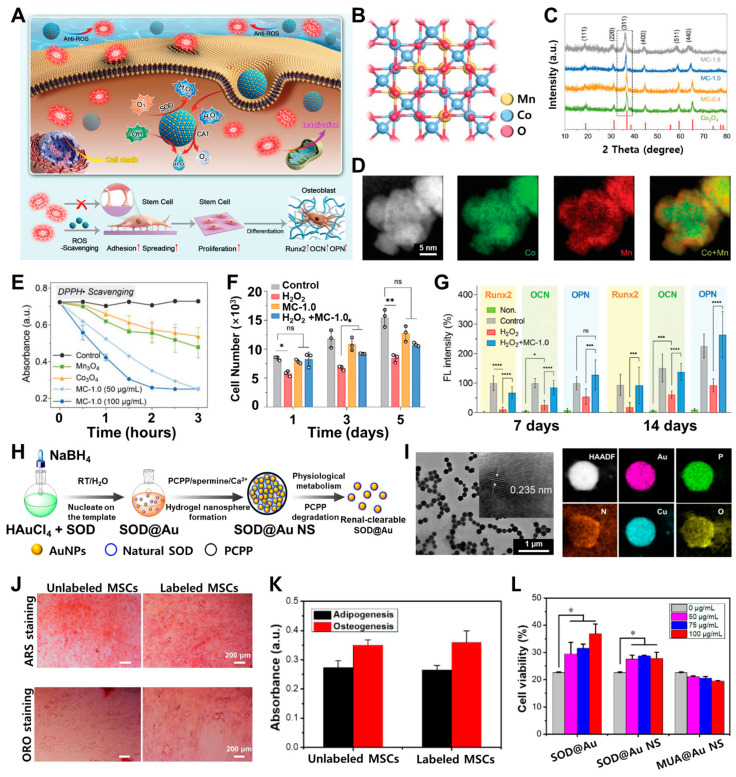
(**A**) Schematic illustrations of Mn-Co_3_O_4_ utilized as antioxidant nanostructures for regulating stem cell fates. (**B**) Crystal model of MC-1.0 which displays the optimized catalytic ROS-scavenging activity. (**C**) X-ray diffraction (XRD) analysis for the Mn-Co_3_O_4_ crystal structures. (**D**) Electron energy-loss spectroscopy (EELS) exhibiting uniformly distributed Co and Mn atoms on the surface. (**E**) Scavenging activities of Mn_3_O_4_, Co_3_O_4_, and MC-1.0 for DPPH radical. (**F**) Quantitative analysis of cell proliferation after the H_2_O_2_ treatment. (**G**) Relative fluorescence intensity of osteogenic markers. (**H**) Schematic diagram of the preparation of SOD-modified gold nanospheres (SOD@AuNS). (**I**) TEM and elemental mapping images of SOD@AuNS. (**J**) Histological analysis of lipid accumulation (ORO) and calcium deposition (ARS) by staining the MSCs, with and without labeling, following adipogenic and osteogenic induction, respectively. (**K**) Quantitative analysis of each differentiation results. (**L**) Cell viabilities of the MSCs labeled with SOD@Au, SOD@AuNS, and MUA@AuNS. The asterisks indicate *p*-values * *p* < 0.05, ** *p* < 0.01, *** *p* < 0.001, **** *p* < 0.0001 and ns represents no significant difference. Reprinted with permission from [101]. Copyright 2022, Wiley Online Library; reprinted with permission from [103]. Copyright 2023, Elsevier.

Tian et al. described a novel strategy involving manganese-atom substitution to modulate the electronic structure of Co_3_O_4_ nanocrystals to enhance their multifaceted catalytic abilities to scavenge ROS [101]. This resulted in the Mn-Co_3_O_4_ material efficiently protecting human MSCs (hMSCs) from ROS-induced damage, reversing apoptotic fates, and rescuing key cellular functions, such as adhesion, spreading, proliferation, and osteogenic differentiation. The performance of hMSCs treated with Mn-Co_3_O_4_ was comparable to that of the hMSCs cultured in standard medium (Figure 3A). The Mn-substituted Co_3_O_4_ materials were synthesized with varying Mn contents and denoted as MC-0.4, MC-1.0, and MC-1.6. Because the MC-1.0 composition exhibited an optimal catalytic ROS-scavenging activity, the subsequent analysis will primarily focus on MC-1.0. All references to the Mn-Co_3_O_4_ materials pertain to the MC-1.0 composition and display spherical and ultrasmall nanoscale morphologies with uniform dispersal (Figure 3B). Moreover, they present clear lattice fringes, corresponding to the lattice planes of Co_3_O_4_. To confirm the uniform crystal structures of Mn-Co_3_O_4_, X-ray diffraction (XRD) was performed, as shown in Figure 3C. The increased substitution of Mn atoms in Mn-Co_3_O_4_ results in a slight shift in the diffraction peaks toward lower diffraction angles compared to pristine Co_3_O_4_. This gradual Mn atom substitution indicates a slight disorder in the Co_3_O_4_ crystalline structure. Subsequently, the incorporation of Mn atoms into the Co_3_O_4_ crystal structure results in longer interatomic distances compared to the native Co-Co bonds, as a consequence of the larger atomic radius of Mn. The electron energy-loss spectroscopy further confirmed (Figure 3D) the homogeneous distribution of both Co and Mn atoms across the surface and edges of the Mn-substituted Co_3_O_4_ material. These findings suggest that the substituted Mn atoms are evenly dispersed throughout the entire crystal lattice of the MC-1.0 composition. Furthermore, DPPH• is a commonly used reagent to assess the free-radical scavenging capacity of biocatalysts. As shown in Figure 3E, the MC-1.0 catalyst also effectively removes DPPH radicals. To further investigate the potential of Mn-Co_3_O_4_ for stem-cell-based therapeutics, researchers systematically evaluated its ability to regulate the fate of the hMSCs in high-ROS environments. Subsequently, the proliferation of the hMSCs under high-ROS conditions was studied to better understand the protective efficacy of MC-1.0 (Figure 3F). The H_2_O_2_-treated hMSCs exhibited the lowest cell counts, indicating that the high oxidative stress had impaired their proliferative capacity. Interestingly, the H_2_O_2_ + hMSCs pretreated with MC-1.0 demonstrated more efficient cell proliferation than the H_2_O_2_-only group. Meanwhile, the data suggest that MC-1.0 had a negligible effect on cell proliferation compared to the control, further confirming the good biocompatibility of the Mn-Co_3_O_4_ material. Previous studies have reported that excessive ROS can impair the osteogenic differentiation potential of the hMSCs [102]. Therefore, this study further investigated the differentiation capabilities of the hMSCs under oxidative stress conditions. It was found that the H_2_O_2_-induced suppression of osteogenic gene expression could be rescued by the addition of MC-1.0. Furthermore, immunofluorescence analyses revealed distinct differences in the signal intensities of the osteogenic markers osteocalcin (OCN) and osteopontin (OPN) between the H_2_O_2_-treated group and other groups (Figure 3G). These results suggest that H_2_O_2_ significantly inhibits the osteogenic differentiation of the hMSCs; however, the addition of MC-1.0 to the H_2_O_2_-containing media can efficiently promote the expression of osteogenic genes in the oxidative stress microenvironment. Collectively, these findings indicate that the MC-1.0 nanomaterial can effectively protect the hMSCs from ROS-induced damage and preserve their critical cellular functions, including adhesion, spreading, proliferation, and differentiation.

A Mn-atom-substituted Co_3_O_4_ nanocrystalline structure and AuNPs can affect anti-inflammation and remove ROS scavengers to protect the potential of the MSCs. Yu et al. developed superoxide dismutase (SOD)-engineered AuNPs as a comprehensive ROS scavenger. SOD is a crucial antioxidant enzyme that neutralizes intracellular ROS and computed tomography (CT) contrast agent for simultaneous protection and imaging tracking of MSCs. It was modified on the surface of the AuNPs and then encapsulated within polyphosphazene nanospheres (NS) (Figure 3H) [103]. This approach aimed to overcome the limited cell membrane penetration and chemical instability of SOD to enhance the survival of MSCs in a harsh inflammatory microenvironment through effective ROS elimination. Further, transmission electron microscopy (TEM) analysis revealed that the engineered SOD@AuNSs possessed a relatively uniform spherical morphology with an average diameter of approximately 270 nm. Elemental mapping further confirmed the presence of gold, phosphorus, nitrogen, copper, and oxygen, which originated from the AuNPs, superoxide dismutase enzyme, and polyphosphazene polymer backbone (Figure 3I). Moreover, the biocompatibility was also confirmed by CCK-8 analysis, maintained over 90% when co-incubated with SOD@AuNSs (Figure 3J). Next, the intracellular localization of SOD@AuNPs in the MSCs was investigated after nucleus staining. The results demonstrated that the SOD@AuNPs were effectively internalized by the MSCs and predominantly distributed within the cytoplasm. This suggests that the SOD@AuNSs can provide feasible cellular contrast signals through stable labeling of MSCs (Figure 3K). Assessing the multipotency of the MSCs after labeling with nanomaterials is crucial for their clinical application. For this purpose, evaluating their ability to differentiate into osteogenic and adipogenic lineages can provide insights into the maintenance of their multipotent potential. As shown in Figure 3L, the SOD@AuNS-labeled MSCs successfully differentiated into adipocytes and osteocytes. Additionally, quantitative assays confirmed that there was no significant difference in the differentiation between the labeled and unlabeled MSCs. Furthermore, protecting stem cells from oxidative stress using metal nanostructures and reversing their apoptotic fates to rescue their critical functions is a promising approach for promoting tissue regeneration. By regulating stem cell fate in microenvironments with excessive ROS, this strategy can effectively advance stem-cell- based therapeutics.

## 3. Carbon-Based Stem Cell Differentiation Approaches and Therapeutics

Over the past several decades, carbon-based nanomaterials have demonstrated a substantial impact within the biomedical fields. These materials possess the capacity to deliver therapeutic agents and enable the visualization of cells and tissues, which are crucial for the treatment and restoration of diseased or damaged tissues [42,58,104]. Carbon-based nanomaterials include fullerenes, carbon nanotubes (CNTs), graphene and its derivatives, GO, nanodiamonds (NDs), and CQDs. Owing to their unique structural dimensions and exceptional mechanical, electrical, thermal, optical, and chemical properties, these materials have attracted significant interest across diverse areas, such as stem cell differentiation and tissue repair applications. Furthermore, surface modification of carbon-based nanomaterials with functional groups can optimize their properties [105]. In addition to their excellent optical characteristics, these materials exhibit high surface areas and exceptional mechanical and electrical properties, making them highly desirable and qualified candidates for theranostic applications [106,107,108]. Importantly, the biological safety of carbon-based nanomaterials—evident from their aqueous stability and interactions with cells and tissues—is a fundamental consideration for their practical biomedical implementation. In this section, we discuss the utility of various carbon-based nanomaterials in regulating stem cell fate and facilitating biomedical applications (Table 2). These include the applications of electrical stimulation and biomolecule delivery to enhance stem cell differentiation and function.

### 3.1. Electrical Signals to Enhance Stem Cell Differentiation

Tissue engineering includes stem cells, scaffolds, and various stimuli such as biochemical and physical stimuli [77,78,113]. For instance, in treating neurodegenerative disorders characterized by neuronal and synaptic loss, guiding stem cell differentiation is crucial for promoting the generation of neurons and synapses through effective induction of neurogenesis [116]. Furthermore, numerous physicochemical cues, including surface roughness, porosity, topography, and chemical composition, have been reported to drive stem cell lineages [117]. Moreover, numerous studies have demonstrated that electrical stimulation is an effective method for promoting the proliferation or differentiation of various stem cell populations [118,119]. This technique offers several advantages, including minimal immune response, controllable stimulation parameters, low tissue damage, ease of implementation, localized induction, and synergistic effects with other differentiation-inducing factors. However, current clinical practices and research studies predominantly rely on external electric fields generated by electrodes or bulky electrical devices to directly induce stem cell differentiation. This invasive method increases the risk of wound-related pain and infection, which is unsuitable for tissue repair applications within the human body. Consequently, there is a clear need for the development of an implantable, cost-effective, and noninvasive stimulation system that aligns with the principles of personalized medicine.

**Figure 4:**
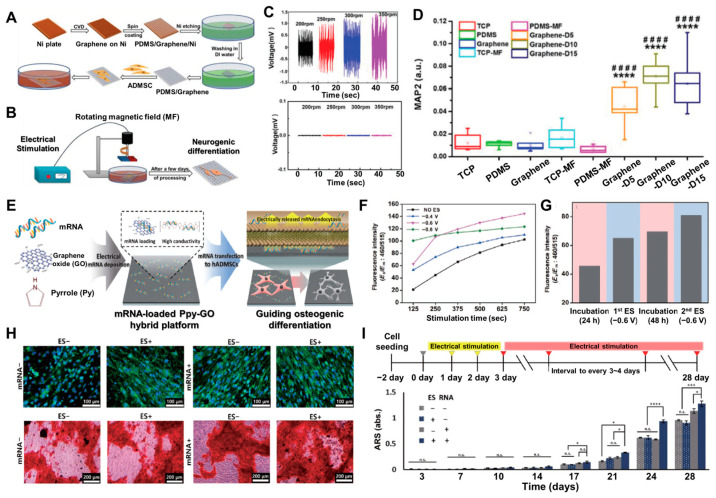
(**A**) Schematic diagram of the characterization and (**B**) electrical stimulation process on graphene film. (**C**) Variations in electrical current or voltage intensity induced by diverse magnetic field (MF) strengths in graphene (upper panel) compared to vehicle control (lower panel). (**D**) Relative fluorescence intensity of MAP2 in the ADMSCs cultured in different substrates. (**E**) Schematic illustration of electrical delivery of mRNA to osteogenic differentiation of the MSCs on the polypyrrole–graphene oxide (PPy–GO)–mRNA hybrid platform. Examination of the electrical modulation of mRNA release using NaFl, based on factors such as (**F**) stimulation duration and (**G**) frequency. (**H**) Assessment of osteogenic differentiation in the MSCs with or without mRNA and electrical stimulation. (**I**) Quantitative analysis of the mineralization in the MSCs during osteogenic differentiation. The asterisks and number sign indicate *p*-values * *p* < 0.05, *** *p* < 0.001, **** *p* and ^####^ *p* < 0.0001. Reprinted with permission from [122]. Copyright 2022, Wiley Online Library; reprinted with permission from [126]. Copyright 2022, Springer Nature.

Graphene-based materials have demonstrated promising applications in the biomedical field, including biosensing, cancer treatment, disease diagnosis, and drug delivery [56,110,111]. The biocompatibility of graphene can be degraded in vivo by human neutrophil peroxidase, and the degradation products exhibit nontoxic side effects. This confirms the biosafety and clinical potential of graphene-based materials. Moreover, in the field of tissue engineering, research has primarily focused on the use of these materials for bone and neural regeneration [120,121]. Guo et al. reported electrical regulation of neural differentiation in adipose-derived mesenchymal stem cells (ADMSCs) through graphene-mediated, wireless, and localized stimulation driven by electromagnetic induction [122]. Various graphene-based scaffold configurations have been investigated and demonstrated that graphene substrates can enhance the neural differentiation of neural stem cells (NSCs), induced pluripotent stem cells (iPSCs), embryonic stem cells (ESCs), and MSCs. However, the beneficial impact of graphene on neural differentiation is typically achieved through the addition of differentiation-promoting factors or is dependent on the specific topological structure of the graphene material. This study reveals that a rotating magnetic field (MF) can directly induce MSCs cultured on graphene films to differentiate into functional neurons without the need for growth factors. This finding offers a promising new strategy for nerve repair using wireless and localized electrical stimulation facilitated by magneto-electric biomaterials (Figure 4A,B). To maintain the structural integrity and facilitate the handling of the graphene film during cell culture experiments, graphene was transferred onto poly (dimethyl siloxane) (PDMS) and denoted as a graphene/PDMS composite material. To evaluate the electromagnetic induction capabilities of graphene and determine its ability to transform magnetic energy into electricity, a model comprising a static graphene/PDMS film was utilized under the influence of a rotating MF. A permanent magnet was selected over a coil because of its superior stability, practicality, and smaller size to generate the same MF strength. PDMS alone did not exhibit any voltage changes during the application of the rotating MF. However, the graphene/PDMS film displayed a significant alternating voltage or current during the MF stimulation, which was sufficient to promote neural differentiation. These results suggest that the graphene film, which acts as a mediator, effectively transformed the magnetic energy into electrical signals through the electromagnetic induction effect, thereby corroborating the initial hypothesis (Figure 4C).

To induce wireless electrical stimulation for neurogenesis, cells cultured on graphene displayed distinct morphological changes when subjected to the rotating MF. After 5 days of treatment, the cells exhibited noticeable cell body shrinkage and transitioned from a fiber-like shape to a more polygonal or rounded morphology. As the duration of the MF exposure was extended, the cells on the graphene film became more elongated and slender, with the gradual emergence of axon-like structures. This phenomenon occurred more rapidly in the group treated with a 10 min electrical stimulation compared to that treated with the 5 min stimulation. These findings suggest that the rotating MF itself did not affect the cytoskeletal morphology and structure of the ADMSCs. However, the induced electrical potential generated on the graphene nanosheets, driven by the electromagnetic effect, altered the fate of the ADMSCs and prompted their neural differentiation. To further elucidate the role of the wireless electrical signal generated on graphene under rotating MF conditions in promoting neuronal differentiation of ADMSCs, immunofluorescence staining was performed. This helped analyze the expression of neural differentiation-related proteins in the ADMSC cultures grown on different substrates, with and without exposure to rotating MF stimulation, over 5, 10, and 15 days. In particular, the mature neuron marker MAP2 increased continuously and stabilized after 10 days of treatment on the graphene film (Figure 4D), which was consistent with the qPCR results. This indicates that continuous wireless electrical stimulation could promote the neural differentiation and maturation of the ADMSCs.

Messenger ribonucleic acid (mRNA), as a key regulator of protein synthesis and cellular signaling pathways, has attracted attention for targeted delivery to specific cells or tissues. mRNA can directly and rapidly initiate protein production and influence various post-transcriptional processes. To alter intracellular gene regulation, cell transfection with exogenous nucleic acid, mRNA, miRNA, and DNA is conducted through lipofection, cell squeezing, sonoporation, or viral method [123,124,125]. However, these methods still encounter several obstacles, including concerns about safety and the risk of compromising cell functionality or viability. Kim et al. reported electrically controlled mRNA delivery using a conductive hybrid film, a polypyrrole–graphene oxide (PPy–GO) film, to promote osteogenic differentiation of hMSCs that can safely and effectively deliver mRNAs to ADMSCs for enhancing osteogenic differentiation (Figure 4E) [126]. Both materials are biocompatible and can support long-term ADMSC culture. The incorporation of GO within the PPy structure facilitates the efficient absorption of mRNAs, and the high conductivity of PPy enables the electrical release of mRNAs from the GO surface. The fabricated PPy–GO hybrid film was then used to load total mRNAs extracted from pre-osteoblasts. It was noted that the osteogenic differentiation of human ADMSCs could be significantly enhanced by the extensive uptake of the total mRNAs from differentiated osteoblasts, which would be electrically released from the hybrid film. Furthermore, a combination of GO and mild electrical stimulation would synergistically promote osteogenic differentiation. As shown in Figure 4F, the electrical release of NaFl exhibited a voltage-dependent pattern. Specifically, the NaFl intensity progressively increased with the increase in voltage levels. However, owing to an initial mRNA burst, the NaFl release at −0.8 V remained negligible for the remainder of the 750 s stimulation period. The NaCl-loaded PPy–GO hybrid film also showed a time-dependent release of the fluorophore in the absence of electrical stimulation. Nevertheless, the fluorescence intensities of the −0.6 V group surpassed those of the No-ES group by 305% and 47% at 125 and 750 s, respectively. Based on the final NaFl intensity and temporal release profile, −0.6 V was used for further electrical release investigations. The researchers repeated the protocol with a 24 h interval of no electrical stimulation (Figure 4G). The NaFl intensity increased over a very brief timeframe, while it increased by only 9.7% after 24 h of incubation without electrical stimulation. Furthermore, the fluorescence lifetime intensity was enhanced when the electrical stimulation was applied to the same hybrid film. This suggests that electrical stimulation is highly effective in facilitating the controlled release of mRNAs into the surrounding medium.

Moreover, the mRNAs were delivered to induce osteogenic differentiation in the hADMSCs. The total mRNAs were electrically immobilized on the hybrid film, and the hADMSCs were then cultured. The experimental conditions were divided into the following four groups: a hybrid platform with or without mRNA and differentiation with or without electrical stimulation. After the differentiation process, the osteogenic differentiation was verified by assessing the expression of OCN and the deposition of hydroxyapatite. Remarkably, the quantification of Alizarin Red S (ARS) staining revealed that the mRNA-loaded film with ES (mRNA^+^/ES^+^) significantly enhanced the mineralization level, which was higher than those obtained with only the hybrid film (mRNA^−^/ES^−^) and the mRNA-loaded film without ES (mRNA^+^/ES^−^) (Figure 4H). To further explore the effectiveness of the mRNA-loaded PPy–GO film combined with electrical stimulation, a time-course study was conducted to identify the specific time points exhibiting the most pronounced differences among the experimental groups. As shown in Figure 4I, the mineralization, measured through ARS staining, became apparent after 14 days of differentiation, particularly in the mRNA^+^/ES^−^ and mRNA^+^/ES^+^ conditions. Notably, the most substantial differences were observed on day 24 of differentiation (DIV 24), where the ARS intensity in the mRNA-loaded groups was higher compared to the other conditions. Interestingly, the ARS intensity in the mRNA-free groups (mRNA^−^/ES^−^ and mRNA^−^/ES^+^) on DIV 28 was similar to the peak levels observed in the mRNA-loaded groups on DIV 24. Collectively, these findings suggest that the electrical stimulation and its biomolecule delivery, such as pre-osteogenic mRNAs, are effective in both the enhancement and acceleration of the differentiation of stem cells, which offers a promising approach for regenerative therapies.

### 3.2. Hydrogel Formation

Natural extracellular matrices (ECMs) have been extensively used as supportive platforms for the adhesion, migration, differentiation, and proliferation of stem cells. However, the poor mechanical properties and unpredictable biodegradation characteristics of natural ECMs significantly limit their potential applications in the biomedical field, underscoring the need for alternative synthetic scaffold materials [127]. As discussed in Section 3.1, conductive scaffolds can facilitate the transfer of electrical signals from the extracellular matrix to cells, thus promoting stem cell differentiation under electrical stimulation. In this regard, carbon-based nanomaterials such as graphene and its derivative, carbon nanotubes (CNTs), and their analogs with high electrical conductivity are promising candidate materials.

Despite the generally reported noncytotoxicity of CNTs toward neurons, several critical concerns must be carefully addressed before their practical application. Primarily, direct exposure to and potential accumulation of CNTs in human tissues may trigger abnormal activation of immune cells and excessive proliferation of fibroblasts, which is a significant concern [128]. One strategy to mitigate these risks is to incorporate CNTs as fillers within a hydrogel matrix. Hydrogel-based materials have demonstrated excellent biocompatibility and show great promise for diverse biomedical applications, including drug delivery, biosensors, and tissue engineering. Consequently, CNT–hydrogel composite materials are often considered promising candidates for combining the electrical conductivity of CNTs with the biocompatibility of hydrogels and can promote regenerative processes [129,130]. However, regarding their impact on neuronal differentiation, the enhanced neuronal excitability induced by the CNTs—which is frequently presumed to support neuronal regeneration—could potentially have adverse effects on the nervous system [131]. For instance, spinal cord injury (SCI) is often associated with increased excitability of motor neurons located below the lesion site, leading to a common and debilitating complication. Therefore, maintaining the homeostatic regulation of neuronal excitability, including the target level of electrical activity, is crucial for processes such as memory storage and activity-dependent neuronal development. In this context, composites of CNTs embedded within a hydrogel matrix may function as biocompatible and conductive scaffolds, potentially minimizing their impact on modulating the intrinsic excitability of neurons.

**Figure 5:**
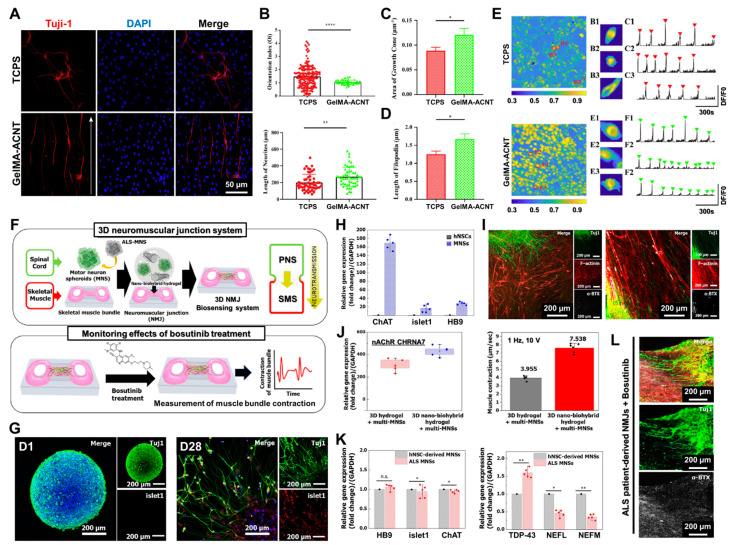
(**A**) Immunofluorescence images for visualization of spiral ganglion neurons (SGNs) after 3 days of culture on TCPS and GelMA–SACNT substrates with the neuronal marker Tuj1. (**B**) Neurite lengths of SGNs cultured on the TCPs and GelMA–SACNT composite substrates. (**C**) Filopodia length and (**D**) growth cone size of SGNs cultivated on the TCPS and GelMA–SACNT substrates. (**E**) Fluorescent imaging of SGNs cultivated on the TCPS and GelMA–SACNT substrates, using the calcium-sensitive dye Fluo-4 AM to record calcium transients, taken after 10 days of culture. (**F**) Schematic of the fabrication and applications of a 3D NMJ system. (**G**) Confocal images of representative marker expression at day 1 and day 28 of neurogenesis in islet1, Tuj1, and Hoechst 33342. (**H**) Quantified results of the ChAT, islet1, and HB9 of the MNSs. (**I**) Confocal images on day 10 of coculture differentiation for muscle bundles with the 3D nano-biohybrid hydrogel using single/multi-MNSs. (**J**) Quantified results of CHRNA7 with 3D and 3D nano-biohybrid hydrogels using multi-MNSs (left panel). Contraction of muscle bundle in the 3D NMJ biosensing system using multi-MNSs upon electrical stimulation (1 Hz, 10 V) (right panel). (**K**) Quantified mRNA results of HB9, islet1, and ChAT on D35 related to MN differentiation between ALS–MNSs and H–MNSs (left panel). TDP-43, NEFL, and NEFM on D35 between ALS-MNSs and H-MNSs (right panel). (**L**) Confocal images of the NMJs treated with 100 μM bosutinib. The NMJs integrated a biosensor using a 3D ALS-nano-biohybrid hydrogel and multi-MNSs. The asterisks indicate *p*-values * *p* < 0.05, ** *p* < 0.01, **** *p* < 0.0001 and ns represents no significant difference. Reprinted with permission from [132]. Copyright 2022, Elsevier; reprinted with permission from [133]. Copyright 2023, Elsevier.

In this regard, Hu et al. demonstrated that patterned substrates fabricated with super-aligned carbon nanotube sheets (SACNTs) can serve as effective topographic scaffolds for regulating and guiding the growth of spiral ganglion neurons (SGNs) [132]. These scaffolds were fabricated by assembling super-aligned carbon nanotubes onto a biocompatible methacrylated gelatin (GelMA) hydrogel. The SACNT sheets offer excellent electrical conductivity and mechanical properties, making them well-suited for application in neurobiological research, particularly in nerve regeneration. By leveraging the aligned configuration of the CNTs and the supportive properties of the GelMA hydrogel, the composite material combines the advantages of the topographical structure of the SACNT sheets with the biocompatibility of the hydrogel. To investigate the regulatory influence of the GelMA–SACNT composite on the SGN growth, immunofluorescence labeling was performed using the early neuronal marker β-III tubulin (β-Tuj1). As shown in Figure 5A, the directional arrow in the image indicates the orientation of the substrate topography. Compared to the tissue culture plates (TCPs) control group, the SGNs cultured on the GelMA–SACNT scaffold exhibited clear directional alignment, growing in parallel with the topographical structure of the composite surface. Moreover, the lengths of the spiral ganglion neurites cultured on the GelMA–SACNT substrate were observed to be significantly greater than those of the SGNs grown on the TCPS control group (Figure 5B). Previous research has demonstrated that migrating neurons develop a growth-cone-like structure at the tips of their leading processes. In vitro studies have indicated that the filopodia and lamellipodia of migrating neurons resemble those found in axonal growth cones, suggesting these structures may play a crucial functional role in neuronal migration. After 3 days of culture, the growth cone areas of the SGNs grown on the GelMA–SACNT were larger than those of the SGNs cultured on the TCPS (Figure 5C). These findings suggest that the GelMA–SACNT scaffold facilitated the growth of the SGNs, likely due to its ability to facilitate cytoskeleton remodeling and axonal regeneration. Additionally, the filopodia within the growth cones, which are important sensory structures for neurite extension, were significantly longer on the GelMA–SACNT substrate compared to the control, although the number of filopodia remained unchanged (Figure 5D). Overall, these findings indicate that the GelMA–SACNT hydrogel scaffold can enhance the growth of SGNs. As shown in Figure 5E, the calcium activities of three representative SGNs were assessed by analyzing the changes in the fluorescence intensities over time. The peak values of the DF/F0 curves were recorded as indicators of the calcium activities. Subsequently, the number of cells exhibiting spontaneous calcium transients was counted across the entire field of view, and the proportion of active cells was calculated. These findings suggest that the GelMA–SACNT scaffold facilitated the growth of the SGNs and promoted synchronization of the calcium transients. Furthermore, the integration of regenerative therapy at the site of injury can potentially optimize its functional capacity and enhance motor rehabilitation. SCIs resulting from traumatic or pathological events often exhibit limited potential for recovery, as they are characterized by the formation of glial scarring, a lack of neurotrophic factors, and disruption of the neural tissue architecture. These factors substantially hinder nerve cell and axon regeneration, thereby impeding the restoration of continuity across the injured spinal cord. Two main approaches have been developed for SCI recovery. The first is regenerative medicine, which involves designing and combining bioactive scaffolds, growth factors/drugs, and cells to induce neural tissue regeneration and motor recovery. The second is rehabilitation, which employs motor training, electrical stimulation, and neurochemical stimulation to leverage the intrinsic plasticity of the nervous system and retrain the remaining uninjured pathways. Therefore, there is a need to develop an integrated approach that combines regenerative therapies to restore spinal cord tissue and rehabilitative interventions to enhance neural plasticity to maximize the potential for functional recovery after an SCI.

Ha et al. have developed a 3D neuromuscular junction (NMJ) system for evaluating the effects of bosutinib treatment using 3D nano-biohybrid hydrogel [133]. NMJs are special connections that promotes signal transfer between motor neurons (MNs) and skeletal muscles. Acetylcholine releases from the synapse at the nerve terminal and binds to the nicotinic acetylcholine receptor (nAChR) in the muscle membrane to form the muscle contract. This binding of acetylcholine triggers muscle contractions. Many researchers have used the current organ-on-a-chip technology to study NMJs by building platforms that mimic human physiological conditions in a lab setting. This has been achieved by increasing the amount of MNs in conjunction with skeletal muscle cells in both 2D and 3D environments. The 3D NMJ systems, in particular, allow for sophisticated modeling of the neuromuscular system by incorporating extracellular matrix proteins, which promote improved biological development. Therefore, the researchers created an innovative 3D NMJ biosensing system by combining CNT-COOH with a 3D nano-biohybrid hydrogel embedded with multiple MNSs. This system was then cocultured with a 3D muscle bundle to observe muscle recovery and movement, particularly for the purpose of drug evaluation (Figure 5F). Immunostaining of class II beta-tubulin (Tuj1), a marker that is common in neurons, was used to assess the system, and it was revealed that the cells grouped together on the first day of differentiation. By day 28 of differentiation, the neurites had extended and formed interconnections (Figure 5G). In addition, the levels of gene expression (SOX1, Nestin, PAX6, islet1, HB9, and ChAT) were assessed before and after MN development (Figure 5H). To construct a 3D biosensing system, they first formed and differentiated muscle bundles in a 3D mold to generate mature myotubes. Subsequently, predifferentiated multi-MNSs (35 × 104 cells per muscle bundle) and CNT-COOH with Matrigel and ECM proteins (fibrinogen and thrombin) were mixed to create a 3D nano-biohybrid hydrogel. Finally, this hydrogel was added to the muscle bundle for coculture. To validate the enhanced growth of new neurons and the formation of NMJs, immunostainings on actin filaments (F-actin; red), α-bungarotoxin (BTX; white), and Tuj1 (a neural marker for neuronal branch/neurite; green) were conducted on the tenth day of coculture differentiation using both a single MNS and multi-MNSs within a 3D nano-biohybrid hydrogel (Figure 5I). Subsequently, the level of expression of the CHRNE gene, responsible for the cholinergic receptor nicotinic epsilon subunit, was measured in the muscle bundle during the development of mature NMJs. Furthermore, an external stimulation of 10 V at a frequency of 1 Hz was applied to the muscle bundle in the 3D NMJ biosensing system (Figure 5J). Because 3D nano-biohybrid hydrogel is advanced and used in the 3D NMJ biosensing system, it facilitates effective ALS medication screening. Thus, this hydrogel was utilized to evaluate the effect of medications on ALS through screening. ALS-MNSs significantly reduced the mRNA levels of the neurofilament light (NEFL) and neurofilament medium (NEFM) chains, serving as markers for nerve fibers. Furthermore, no notable differences in the mRNA levels of islet1, ChAT, and HB9 between ALS-MNSs and H-MNSs (Figure 5K) were observed. Additionally, the 3D ALS nano-biohybrid hydrogel treated with bosutinib exhibited a greater number of neurite outgrowths compared to the untreated control (Figure 5L). Therefore, carbon-based materials combined with the hydrogel offers a promising approach for integrating regenerative therapies and rehabilitation strategies for stem-cell-based recovery.

## 4. Other Nanomaterials for Stem Cell Differentiation and Therapeutics Applications

Over the past decades, various strategies have been explored to protect growth factors from in vivo inactivation or degradation. These approaches aim to enable the precise, localized, and temporally controlled delivery of growth factors. These strategies include the use of hydrogels, polymer matrices, and liposomes. However, the harsh chemical processes, such as crosslinking and conjugation reactions, involved in these strategies may potentially compromise the integrity of the growth factors. More recently, inorganic nanoparticles, particularly those with internal void spaces and porous structures, have been explored as drug delivery platforms owing to their high loading capacity [134,135,136]. In addition, photoresponsive nanomaterials have garnered significant attention in the biomedical field owing to their ability to remotely modulate material properties in intrinsic spatial and temporal domains without physical interaction. Likewise, the development of self-assembled nanoscale materials that integrate both inorganic and organic components has rapidly produced unique hierarchical structures. These hierarchical structures provide unprecedented combinations of technologically appealing functionalities that are frequently unachievable in traditional composites and other materials [137,138]. This section explores the potential of various nanomaterials in controlling the delivery of biomolecules and promoting stem cell differentiation. It also reviews related previous studies on this topic (Table 3). Furthermore, this section specifically discusses the application of MOFs, ZIFs, and upconversion nanoparticles (UCNPs) for stem-cell-based therapies.

### 4.1. Metal–Organic Framework

One of the primary advantages of nanomaterials in biomedical applications is their ability to serve as versatile platforms for targeted delivery of bioactive cues, such as growth factors, genes, and small molecules, to stem cells [149]. By encapsulating or conjugating signaling moieties to the surface of nanomaterials, researchers have achieved improved control over stem cell differentiation. This approach has the potential to direct stem cells toward specific cell types needed for tissue repair and regeneration. This level of precision and control over stem cell differentiation is crucial for the development of effective stem-cell-based therapies, as it can help overcome the challenges associated with the low efficiency and poor survival of transplanted stem cells. For instance, MOFs—a class of crystalline porous materials composed of metal ions/clusters and organic ligands—offer unique advantages such as an exceptionally high surface area, tailorable shape, and uniform pore sizes. Fabricating organic–inorganic hybrid nanomaterials enables MOFs to serve as versatile reservoirs for both hydrophobic and hydrophilic drug molecules [150]. Furthermore, their weak coordination interactions contribute to their excellent biodegradability. Notably, zinc ions have been reported to promote osteogenic differentiation in MSCs. This suggests that Zn-based MOFs, such as zeolite imidazolate framework-8 (ZIF-8), may possess significant advantages for delivering biomolecules to facilitate stem cell regeneration [139,151].

Neural stem cell (NSC) therapy, which aims to replace lost and damaged neurons, has been proposed as a potential treatment for stroke. However, the therapeutic potential of NSC-based treatments is limited by the low rate of neuronal differentiation observed in these cells. The overexpression of miR-124 can increase the number of newly formed neurons by suppressing the expression of non-neuronal genes [152]. Studies have shown that neuron-specific miR-124 is important in regulating neuronal death caused by ischemic stroke, and its overexpression can improve functional recovery in animal models of ischemic stroke [153]. However, the clinical application of miR-124-based therapies is limited because the naked miRNA is unstable and poorly absorbed by cells owing to its negative charge. Conventional organic nanoparticles, such as liposomes, are commonly used as nonviral vectors for miRNA delivery; however, they are often associated with significant immunogenicity, leading to tissue damage and inflammation. In contrast, MOFs have been demonstrated as favorable carriers for miRNA delivery owing to their functional surface properties, high surface area, and self-assembly structure.

**Figure 6:**
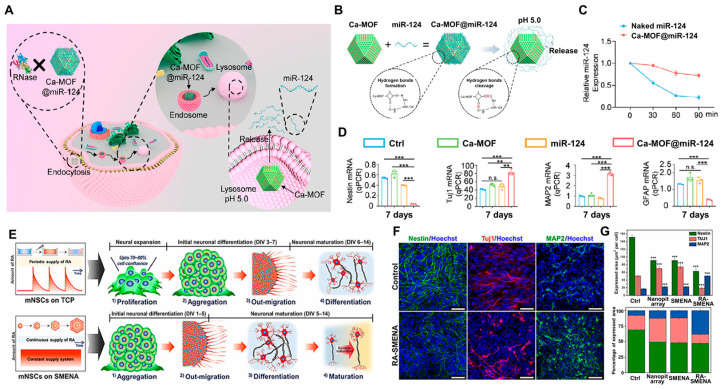
(**A**) Schematic of the delivery and uptake of miR-124 loaded onto Ca-MOF nanoparticles within NSCs. (**B**) Illustrations of the embedding strategy of miR-124 onto the surface of Ca-MOF by hydrogen bonds between the −NH_2_ and −OH groups and the rapid cleavage of the hydrogen bonds upon exposure to a low pH. (**C**) Protective effect of Ca-MOF@miR-124 nanoparticles from nuclease degradation. (**D**) The results of the quantitative PCR analysis performed to evaluate the expression of various neural markers in differentiated NSC groups cultured with Ca-MOF, miR-124, or Ca-MOF@miR-124 nanoparticles over different time points. (**E**) Schematic of the different differentiation mechanisms between the conventional supply method and autonomous stem cell differentiation (SMENA). (**F**) Immunocytochemical analysis of the neuronal differentiation for each treatment group at DIV 14 and (**G**) quantitative analysis and relative quantification of the protein expression. The asterisks indicate *p*-values ** *p* < 0.01, *** *p* < 0.001 and ns represents no significant difference. Reprinted with permission from [154]. Copyright 2022, American Chemical Society; reprinted with permission from [155]. Copyright 2022, American Association for the Advancement of Science.

Yang et al. used an MOF (Ca(C_4_O_4_)·(H_2_O), Ca-MOF), a biocompatible nanoparticle-based delivery system for miR-124. This approach was designed to enhance the neuronal differentiation of NSCs and to integrate this delivery system with NSC therapy for the treatment of ischemic stroke (Figure 6A) [154]. The formation of hydrogen bonds between the amino groups of miR-124 and the hydroxyl groups on the surface of Ca-MOFs enabled the efficient loading of miR-124 onto the Ca-MOF nanoparticles. This approach facilitated the effective delivery and protection of miR-124 from degradation. Ca-MOF@miR-124 nanoparticles were simply prepared by mixing appropriate amounts of miR-124 and Ca-MOF nanoparticles in an aqueous medium. The hydrogen bonds were quickly disrupted in acidic conditions, enabling the release of miR-124 and its subsequent internalization into cells after being taken up into endosomes or lysosomes. Enhancing the stability and protection of miR-124 is critical for advancing the clinical application of RNA delivery. Anchoring miR-124 to Ca-MOF preserves its native structure and prevents degradation during cellular uptake. This approach is crucial for effective miR-124 delivery, addressing the inherent challenges associated with the properties of miR-124. As shown in Figure 6B, miR-124 was attached to the surface of Ca-MOFs through hydrogen bonding between the amino and hydroxyl groups, forming Ca-MOF@miR-124 complexes. This strategy could protect miR-124 from nuclease degradation and enhance its uptake by NSCs. To confirm stability under nuclease degradation conditions, the intensity of the Ca-MOF@miR-124 group was only 26% lower after 90 min of nuclease exposure, while that in the naked miR-124 group was almost completely gone after just 60 min. These results demonstrate that the Ca-MOF@miR-124 nanoparticles effectively protected miR-124 from nuclease degradation (Figure 6C). Subsequently, to assess the acceleration of NSC neuronal differentiation, NSCs were cocultured with Ca-MOF nanoparticles, miR-124, or Ca-MOF@miR-124 nanoparticles for 5 and 7 days. As shown in Figure 6D, the expression of the NSC marker Nestin in the Ca-MOF@miR-124 nanoparticle group was lower compared to those of the control, Ca-MOF nanoparticle, and miR-124 groups on day 5. This study presents a promising combination therapy for ischemic stroke, utilizing Ca-MOF@miR-124 as a highly effective and biocompatible nanoparticle-based delivery system for miR-124. Consequently, these nanocarriers facilitate the directed neuronal differentiation of transplanted neural stem cells, thereby enhancing their therapeutic potential. Similarly, on day 5, the expression of MAP2—another neuronal marker—was higher in the Ca-MOF@miR-124 nanoparticle group compared to the control, Ca-MOF nanoparticle, and miR-124 groups. However, the mRNA expression of GFAP—a glial marker—was significantly lower in NSCs treated with Ca-MOF@miR-124 nanoparticles compared to control NSCs and Ca-MOF-treated NSCs on day 5. This work describes a promising combination therapy for ischemic stroke that employs Ca-MOF@miR-124 as a highly effective and biocompatible nanoparticle-based delivery system for miR-124. As a result, useful nanocarriers promote the directed neuronal differentiation of transplanted NSCs, enhancing their therapeutic potential.

Similarly, Cho et al. used MOFs to drive stem cell differentiation through the slow release of retinoic acid (RA) molecules over extended timescales and addressing concerns about the need for replenishing differentiation factors [155]. However, nanoparticles can potentially induce cytotoxicity in stem cells when in direct contact. To mitigate this adverse effect, researchers have developed a platform incorporating nanoscale wells or pits to encapsulate MOF nanoparticles. This single metal–organic framework-embedded nanopit arrays (SMENA) were fabricated using interference lithography, which allowed precise control over the lithography parameters to create sufficiently deep nanopits. This design physically separates the MOF nanoparticles from the stem cells, ensuring that each nanopit contains only one nanoparticle, thus preventing direct contact between the nanoparticles and the stem cells. This spatial segregation, assuming the homogeneous incorporation of one nanoparticle per nanopit, prevents direct contact between the nanoparticles and the stem cells. The improved protection and slow release of the platform enable the autonomous differentiation of stem cells into fully mature neurons, thereby significantly diminishing the risks associated with manually replenishing RT for differentiation induction. To demonstrate the concept, a mouse NSC line capable of differentiating into neurons under RA treatment was selected as the model system. For the control groups, the NSCs were first exposed to the RA treatment after 2 days of culture to allow for metabolic stabilization. RA is highly unstable under standard cell culture conditions and, therefore, requires fresh preparation every 2 days during medium changes throughout the differentiation period. In contrast, the experimental procedure for the NSCs cultured on single RA-containing MOF-embedded nanopit arrays was markedly different from the conventional approach (Figure 6E) Owing to the continuous release of RA from MOF, only a standard growth medium was used and replaced every 2 days without the need for external RA addition. In contrast, the expressions of Tuj1 and MAP2 were higher in the RA-SMENA group than in the control group, respectively. By DIV 14, the expression of MAP2 had increased in the RA-SMENA group compared to the control, while the expression of Nestin and other premature neuronal markers decreased significantly (Figure 6F,G). This platform is highly effective at rapidly maturing NSCs into neurons with high efficiency. Collectively, these results highlight the potential of the MOF-based embedding of biomolecules as a powerful platform for stem cell differentiation and in therapeutic applications with precise control over the delivery of inductive factors.

### 4.2. Upconversion Nanoparticles

Upconversion nanoparticles (UCNPs), a unique class of inorganic phosphors, can absorb near-infrared (NIR) light. Moreover, it can convert, through sequential photon absorption, into ultraviolet-visible emissions. These UCNPs possess several advantageous properties, including excellent optical stability, low toxicity, deep tissue penetration, biocompatibility, minimal background fluorescence interference, and high imaging sensitivity [156,157]. Consequently, UCNPs have significant potential for applications in stem cell differentiation and therapeutic interventions [158]. For instance, multifunctional UCNPs have been utilized as nanoscale fluorescent probes to monitor the localization and distribution of MSCs over extended periods. Additionally, UCNPs have been incorporated into cell culture substrates to modulate the interactions between MSCs and the substrate, thereby enabling the control of multilineage stem cell differentiation through NIR intensity variations. Furthermore, UCNPs have been developed as nanocarriers capable of the NIR-triggered release of biomolecules, enabling the regulation of MSC differentiation. Moreover, UCNPs have been used as nanoscale probes to detect osteogenic differentiation of stem cells by monitoring changes in fluorescence between UCNPs and fluorescein isothiocyanate (FITC).

**Figure 7:**
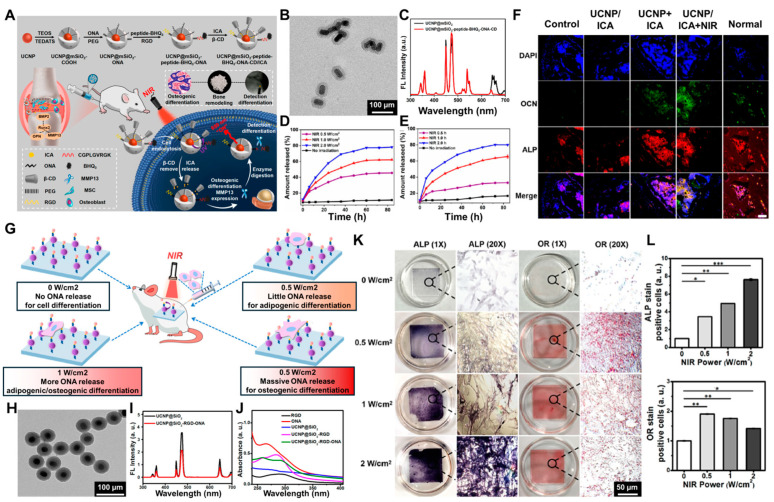
(**A**) Schematic of the functionalized UCNP platform. This platform is designed for NIR light-controlled and real-time monitoring of osteogenic differentiation in MSCs. The platform facilitates precise tracking and modulation of osteogenic differentiation processes, aiding in the development of targeted therapies for osteoporosis. Characterizations of the UCNP platform. (**B**) TEM image of UCNP@mSiO2-peptide-BHQ3-ONA-CD and (**C**) spectrum upon irradiation at 980 nm with 1 W/cm^2^. (**D**) Quantification of the NIR-mediated release of ICA from the UCNP nanocomplex at different NIR power levels (0, 0.5, 1, and 2 W/cm^2^) and (**E**) irradiation durations at 1 W/cm^2^. (**F**) Immunofluorescence analysis of MSCs following 10 days of culture, evaluating the expression of proteins associated with osteogenic differentiation markers. (**G**) Schematic of the NIR stimulation triggering the release of ONA, enabling the control of cell adhesion, spreading, and multilineage differentiation of MSCs on the UCNP-based substrate under different NIR irradiation intensities. (**H**) TEM image and (**I**) fluorescence emission of the UCNP@SiO_2_-RGD-ONA nanoparticles under 980 nm NIR irradiation. (**J**) UV-vis absorption spectra of RGD, ONA, UCNP@SiO_2_, UCNP@SiO_2_-RGD, and UCNP@SiO_2_-RGD-ONA. (**K**) Histological analysis to evaluate the osteogenic and adipogenic differentiation of MSCs cultured on the UCNP substrate following exposure to NIR irradiation at different intensities, after 7 days of induced differentiation. (**L**) Quantification of positive cells for different powers of NIR. The asterisks indicate *p*-values * *p* < 0.05, ** *p* < 0.01, *** *p* < 0.001. Reprinted with permission from [159]. Copyright 2022, American Chemical Society; reprinted with permission from [160]. Copyright 2022, American Chemical Society.

In terms of stem cell differentiation and therapeutic applications, Yan et al. developed a functionalized UCNP-based nanoplatform for NIR light-controlled and real-time monitoring of osteogenic differentiation in MSCs. This system enables real-time monitoring of differentiation and aims to advance therapeutic strategies for osteoporosis (OP) [159]. The authors engineered UCNP-based light-responsive nanoplatforms that enable NIR triggered intracellular delivery of icariin (ICA). ICA is a natural compound with potent antioxidant and phytoestrogen properties. It has been shown to inhibit bone loss in osteoporosis (OP) (Figure 7A). The Tm/Er-doped NaYF4 core–shell UCNPs were first synthesized and coated with mesoporous silica to load ICA. The surface of UCNP@mSiO2 was then modified with N-propyl ethylenediamine triacetic acid to conjugate a photocaged linker, PEG linker, β-cyclodextrin cap, and peptides targeting the Arg-Gly-Asp motif and matrix metalloproteinase 13 (MMP13), resulting in the formation the UCNP nanoplatform. Finally, the ICA was loaded into the porous silica to obtain the UCNP nanocomplex, as shown in Figure 7B. The photoluminescence of UCNP@mSiO2 and UCNP@mSiO2-peptide-BHQ3-ONA-CD under 980 nm NIR light excitation exhibited a strong emission spectrum (Figure 7C). This spectrum showed that the upconverted UV/visible light could trigger UV-sensitive photochemical reactions, thereby triggering the release of ICA and simultaneously detecting MMP13 activity. To assess the NIR-mediated ICA release, the UCNP nanocomplex was dispersed in a PBS buffer and exposed to different NIR irradiation intensities. As shown in Figure 7D, the ICA was released without any NIR irradiation. The release rate of ICA from the UCNP nanocomplex increased after 1 h of NIR irradiation at 1 W/cm^2^. The release rate further rose to 78.13% after 1 h of irradiation at 2 W/cm^2^, followed by 84 h of incubation. This demonstrates the effective NIR-triggered release of ICA from the UCNP nanocomplex. Additionally, a time-dependent NIR-mediated release experiment showed that ICA release remained low in the absence of NIR irradiation but increased progressively with extended NIR exposure times (Figure 7E). These findings suggest that the release of the ICA is dependent on NIR irradiation, with longer exposure times resulting in higher release amounts. After confirming the effective intracellular delivery of UCNPs to MSCs, the authors conducted experiments to investigate the NIR-triggered release of ICA from the UCNP nanocomplex and its potential to modulate the osteogenic differentiation of MSCs in vitro. In particular, the upconverted UV light generated by the UCNPs under NIR excitation cleaved the photocaged linker and removed the capping β-cyclodextrin, releasing the ICA cargo from the UCNP nanocomplex. To confirm the NIR-triggered release of the ICA to control stem cell differentiation, immunofluorescence staining was used to further verify the osteogenic differentiation of MSCs after NIR irradiation treatment (Figure 7F). Compared to the control, UCNP, and UCNP/ICA groups, the UCNP/ICA + NIR group exhibited high expression of proteins associated with osteogenic differentiation, such as OPN, Runx2, MMP13, and BMP-2, after 7 days of continuous culture. This indicates that the NIR treatment and release of ICA from the UCNP nanocomplex effectively induced the osteogenic differentiation of MSCs. Particularly, the expression of OPN in the UCNP/ICA + NIR group was more than three times higher that of the UCNP/ICA group without NIR treatment. The expression levels of Runx2, MMP13, and BMP-2 were higher compared to the UCNP/ICA group. These results demonstrate that the NIR-mediated ICA release could effectively induce osteogenic differentiation of MSCs, highlighting the ‘light control of cell differentiation’ using the UCNP nanoplatform.

Furthermore, recent studies have shown that UCNPs can be utilized to engineer substrates capable of modulating cell adhesion and differentiation through near-infrared light stimulation. Guo et al. reported the use of UCNP-based substrates to regulate the activation of adhesion-promoting RGD peptides, which in turn modulates cell adhesion and multilineage differentiation of MSCs through NIR light stimulation [160]. In these systems, core–shell UCNPs were synthesized and coated with silica to enable the conjugation of RGD peptides via an NHS-PEG-MAL linker. Additionally, the photocleavable molecule 4-nitrobenzoic acid reacted with the aspartic acid (Asp) residue of the RGD peptide, temporarily inactivating the cell adhesion properties of the RGD peptide (Figure 7G). To fabricate the UCNP-based substrate, core–shell UCNPs were synthesized. In addition, the UCNPs exhibited strong UV emission under 980 nm NIR irradiation, indicating their potential for photocleavage applications. Subsequently, the UCNPs were coated with a silica shell and functionalized with the RGD peptide using an NHS-PEG-MAL linker. Moreover, the photocleavable oligonucleotide (ONA) was coupled to the aspartate residue of the RGD peptide, yielding the UCNP@SiO2-RGD-ONA nanostructure. Transmission electron microscopy (TEM) revealed that the UCNP@SiO2-RGD-ONA nanoparticles had an average diameter of approximately 50 nm (Figure 7H). Furthermore, the UCNP@SiO2-RGD-ONA demonstrated strong upconverted UV emission when illuminated with 980 nm NIR light (Figure 7I), similar to the UCNP@SiO2 counterpart. To confirm the successful fabrication of the UCNP@SiO2-RGD-ONA construct, UV-vis analysis was performed, showing effective conjugation of ONA and RGD to the UCNP surface (Figure 7J). Subsequently, to investigate the effect of the RGD peptide on cell adhesion, UCNP substrates were prepared with or without RGD modification. The UCNP substrate without RGD modification showed limited cell adhesion, whereas the substrate modified with the RGD peptide supported the adhesion of a substantial number of MSCs after 24 h of culture. Following these observations, the researchers investigated whether MSCs, exhibiting different morphologies due to various light irradiation conditions, could undergo osteogenic and adipogenic differentiation. Alkaline phosphatase (ALP) and oil red O (OR) staining were performed in each sample to evaluate the multilineage functions on each substrate. As shown in Figure 7K,L, MSCs cultured on the substrate subjected to low-power NIR irradiation exhibited light ALP staining and strong OR staining, whereas those on the high-power NIR-treated substrate exhibited a robust ALP staining and a faint OR staining. This indicates that low-power NIR irradiation promotes the adipogenic differentiation of MSCs, while high-power NIR irradiation favors their osteogenic differentiation. Collectively, these findings demonstrate that the UCNP-based substrate can regulate the multilineage differentiation of MSCs by modulating their adhesion and spreading in response to varying NIR light intensities. Consequently, Figure 7 presents a new approach for the noninvasive monitoring of stem cell differentiation and regulation of cell adhesion using NIR light. The proposed method is a powerful tool for light-controlled therapeutic strategies in various stem-cell-based treatments.

## 5. Conclusions and Future Perspectives

In recent years, significant advancements in nanomaterials have substantially impacted the field of biomedicine. These nanomaterials, which include metal-based, carbon-based, and nanoframework-based nanomaterials, have improved the ability of scaffolds to mimic the complex properties of bone and neural tissues. This has resulted in a microenvironment conducive to cell proliferation, adhesion, and differentiation. Furthermore, nanomaterials have demonstrated the ability to modulate the spatial and temporal release of key substances involved in stem cell reprogramming and differentiation, thereby improving the efficiency and safety of these processes. This review emphasizes how nanomaterials may interact with stem cells to guide their differentiation into different cell types, such as osteogenic, neurogenic, and adipogenic cells. This is achieved through the modulation of specific cellular signaling pathways and differentiation-related factors, as well as the regulation of cell proliferation and adhesion. In the future, intracellular regulation, external stimulation, and the control of cellular adhesion will likely represent unique mechanisms through which nanomaterials exert their effects. Nanomaterials with specific properties and configurations have demonstrated the ability to guide stem cell differentiation into desired lineages. However, the precise mechanisms underlying this phenomenon are not well understood, as the existing literature has not thoroughly explored the mechanisms by which nanomaterials promote stem cell differentiation. Further research is therefore needed to elucidate the complex interplay between the unique physicochemical characteristics of nanomaterials and their subsequent impact on the biological processes governing stem cell fate. This includes investigating how nanomaterials can modulate key signaling pathways, gene expression profiles, and cellular biomechanics to drive stem cell differentiation toward specific lineages. Additionally, to enhance the differentiation of stem cells toward specific lineages, the development of novel nanomaterials with appropriate nanobio-interfaces and optimized physical, biochemical, and biomechanical cues is warranted. Such advanced nanomaterials could provide more targeted and effective strategies for stem-cell-based therapies and tissue engineering applications. In the future, individualized therapeutics may employ nanoparticles to regenerate severely injured tissues, leveraging nanotechnologies to modulate stem cell behavior.

## Figures and Tables

**Table 1:** Metal-based stem cell differentiation approaches and therapeutics.

Nanomaterials	Lineages	Cells	Mechanism of Actions	Therapeutic Applications	Ref.
AuNPs	Osteogenic differentiation	MSCs	ROS scavenging	Periodontitis	[76]
CaFO NPs (calcium folate)	Neuronal differentiation	Mouse embryonic-derived NSCs	Decomposition into Ca^2+^ and folic acid	Alzheimer’s disease	[77]
SPIO-AuNPs	Neuronal differentiation	PC-12	Dynamic magnetic field to Ca^2+^ influx	Alzheimer’s disease	[78]
AuNPs	Neuronal differentiation	Embryonic-derived NPCs	GFAP barriers from activated astrocytes	Spinal cord injury	[79]
L/D-Au nanocluster films	Adipogenic/ Osteogenic differentiation	MSCs	Chirality at cluster scale to control cellular behaviors	Functional maintenance in organisms	[80]
Chitosan-AuNPs	Neuronal differentiation	MSCs	Strengthen MSC colony formation	Neurodegenerative diseases	[81]
Chiral AuNPs	Neuronal differentiation	Mouse neural NSCs	CPL illumination to direct differentiation	Alzheimer’s disease	[82]
CeO2 NPs	Osteogenic differentiation	MC3T3-E1	Activating the Wnt pathway	Bone metabolic disease	[83]
AgNPs-Ca(OH)_2_	Osteogenic differentiation	MSCs	Upregulation of TGF-β1	Necrosis in immature teeth	[84]

**Table 2:** Carbon-based stem cell differentiation approaches and therapeutics.

Nanomaterials	Lineages	Cells	Mechanism of Actions	Therapeutic Applications	Ref.
Graphene oxide	Chondrogenic differentiation	MSCs	Activating osmosensitive receptor	Cartilage repair	[109]
Graphene oxide QDs	Osteogenic differentiation	MSCs	Promoting mitochondrial dynamics	Bone defects	[110]
3DGp/CNT scaffolds	Osteogenic differentiation	MSCs	Upregulation of BMP pathway	Dental clinical engineering	[111]
CNTs	Neuronal differentiation	NSCs	Enhancing cellular attachment and communication	Neurological diseases and injuries	[112]
Carbon nanofibers	Neuronal differentiation	MSCs	Increasing cellular connection	Neural tissue regeneration	[113]
Silk fibroin/ carbon nanofiber scaffolds	Cardiomyogenic differentiation	iPSCs	Mimicking mechanical/physical properties of cardiac tissue	Heart failure	[114]
CNTs	Neuronal differentiation	NSCs	Regulating focal adhesion, calcium ion channels/ PI3K-AK pathways	Cortical injury	[115]

**Table 3:** Various nanomaterials-based stem cell differentiation and therapeutics applications.

Nanomaterials	Lineages	Cells	Mechanisms	Therapeutic Applications	Ref.
Stem cell membrane/ZIF-8	Osteogenic differentiation	MSCs	Improve the targeted internalization of nanoparticles	Bone regeneration	[139]
Dexamethasone/ZIF-8	Osteogenic differentiation	MSCs	Activation of PI3K-Akt signaling pathways	Bone regeneration	[140]
ZIF-8/GelMA hydrogel	Neuronal differentiation	DPSCs	Activation of MAPK signaling pathway	Spinal cord injury	[141]
Porous carbon nanozyme/HOFs	Neuronal differentiation	NSCs	Oxidative stress resistance, drug carrier	Alzheimer’s disease	[142]
Ce/Sr dual-loaded bio-MOF	Osteogenic differentiation	MSCs	Restore mitochondrial dynamics and normalize senescent MSCs	Osteoporotic fracture	[143]
Zn/Co-MOF/β-TCP scaffolds	Osteogenic/ chondrogenic differentiation	MSCs	ROS scavenging	Osteoarthritis	[144]
UCNPs-F127@Cur	Glioma stem cells	NIH3T3	Suppressing the Wnt-β-catenin and Jak-Stat pathways	Glioblastoma	[145]
PLLA nanofiber	Neural differentiation	NSCs	Piezoelectric charge to topographical stimulation	Neural tissue repair	[146]
Single-chain atomic crystal	Neural differentiation	ESCs	Supporting adhesion and growth	Neurodegenerative disease	[147]
Graphene derivatives/CeO_2_-nanoparticle-containing hydrogels	Neuronal differentiation	NSCs	Self-assembly of graphene oxide sheets incorporating a reducing agent and CeO_2_ nanoparticles	Neuroregenerative cell therapies	[148]

## Data Availability

Not applicable.
